# Metal-organic framework-based photodynamic combined immunotherapy against the distant development of triple-negative breast cancer

**DOI:** 10.1186/s40824-023-00447-x

**Published:** 2023-11-24

**Authors:** Xiaoyan Liang, Min Mu, Bo Chen, Rangrang Fan, Haifeng Chen, Bingwen Zou, Bo Han, Gang Guo

**Affiliations:** 1 Department of Biotherapy, Cancer Center and State Key Laboratory of Biotherapy, West China Hospital, Sichuan University, Chengdu, 610041 China; 2 Department of Neurosurgery, West China Hospital, Sichuan University, Chengdu, 610041 China; 3 School of Pharmacy, and Key Laboratory of Xinjiang Phytomedicine Resource and Utilization, Ministry of Education, Shihezi University, Shihezi, 832002 China

**Keywords:** Triple-negative breast cancer, Metal-organic frameworks, Immunogenic cell death, Photodynamic therapy, Immunotherapy

## Abstract

**Background:**

Triple-negative breast cancer (TNBC) is an aggressive, metastatic and apparently drug-resistant subtype of breast cancer with a higher immune response compared to other types of breast cancer. Photodynamic therapy (PDT) has been gaining popularity for its non-invasive nature, minimal side effects, and spatiotemporally controlled benifits. The use of metal-organic frameworks (MOFs) loaded with programmed death-ligand 1 inhibitors (iPD-L1) offers the possibility of combining PDT with immunotherapy.

**Method:**

Here, we construct PCN-224, a MOFs with good biocompatibility and biodegradability for the delivery of the PD-L1 small molecule inhibitor BMS-202 to achieve a synergistic anti-tumor strategy of PDT and immunotherapy. Hyaluronic acid (HA) modified PEG (HA-PEG) was synthesized for the outer layer modification of the nanocomplex, which prolongs its systemic circulation time.

**Results:**

In vitro cellular experiments show that the nanocomplexes irradiated by 660 nm laser has a strong ability to produce singlet oxygen, which effectively induce PDT. PDT with strong immunogenicity leads to tumor necrosis and apoptosis, and induces immunogenic cell death, which causes tumor cells to release danger associated molecular patterns. In combination with iPD-L1, the combination therapy stimulates dendritic cell maturation, promotes T-cell activation and intratumoral infiltration, and reshapes the tumor immune microenvironment to achieve tumor growth inhibition and anti-distant tumor progression.

**Conclusions:**

MOFs-based nano-systems as a platform for combination therapy offer a potentially effective strategy for the treatment of TNBC with high metastatic rates.

**Graphical Abstract:**

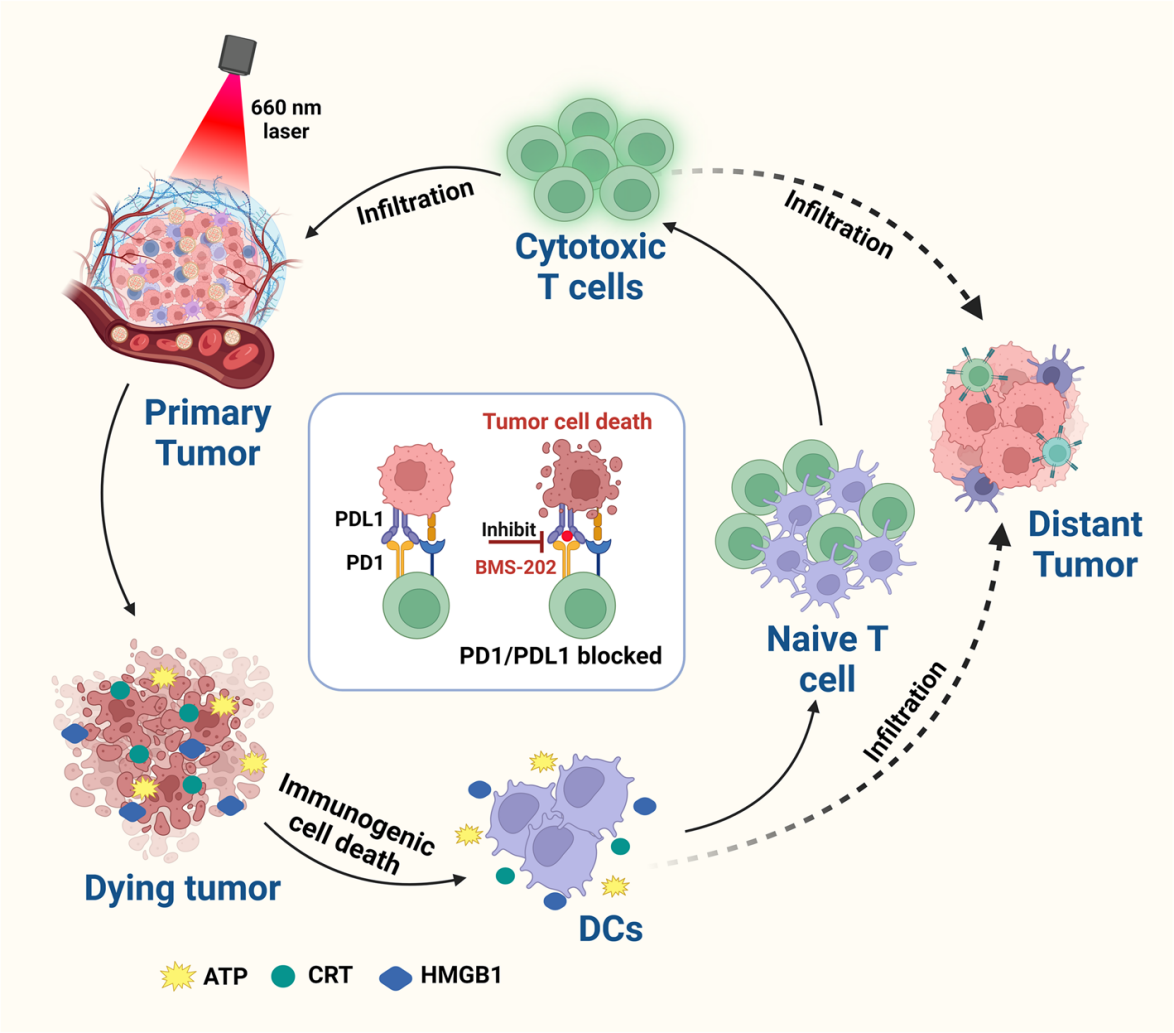

**Supplementary Information:**

The online version contains supplementary material available at 10.1186/s40824-023-00447-x.

## Introduction

The Global Cancer Statistics 2020 report published by the World Health Organization states that female breast cancer has surpassed lung cancer as the most common cancer, with an estimated 2.3 million new cases (11.7%) and approximately 6.9% of all new deaths from cancer [1, 2]. Triple negative breast cancer (TNBC) [3] is a breast cancer that is negative for estrogen receptor, progesterone receptor and proto-oncogene HER2, accounting for 15%-20% of all breast cancers. It is highly resistant, heterogeneous and aggressive, and is often associated with pulmonary or brain metastases [4–7]. Immunotherapy is a new pillar in the treatment of other cancers and has shown exciting results in some TNBC patients. Studies have shown that about 20% of TNBC patients have positive PD-L1 expression, which is significantly higher than non-TNBC patients [8], suggesting that TNBC patients are suitable for PD-1/PD-L1 immunotherapy [9, 10].

The tumor microenvironment (TME) is a highly complex and heterogeneous environment which results in tumor invasion and metastasis, treatment resistance, immunosuppression and evasion of immune surveillance [11–14]. In TME, the binding of PD-L1 on tumor cells to PD-1 on tumor-infiltrating T cells inhibits the activation and proliferation of T cells, enabling immune escape of cancer cells [15–19]. A significant proportion of clinical patients exhibit a low immune response, making immunotherapy ineffective. Therefore, there is an urgent need for additional strategies to expand the patient population that can benefit from anti-PD-1/PD-L1 therapy.

Photodynamic therapy (PDT) [20–24] is highly immunogenic and has been found to cause tumor cell death in an immunogenic manner [25–27]. Immunogenic cell death (ICD) is a specific form of apoptosis that allows a well-immunized host to trigger specific immune responses against dead cell-associated antigens [28]. Reactive oxygen species (ROS) [29–31] produced by PDT not only promote tumor cell death but also trigger endoplasmic reticulum stress, causing calreticulin (CRT) exposure, which induces ICD and release of pro-inflammatory cytokines and DAMPs from tumor cells [32, 33]. The released DAMPs stimulate the maturation of dendritic cells (DCs), and subsequently mature DCs bearing tumor-specific antigens migrate to lymph nodes [34] and activate T cells; activated T cells migrate and infiltrate into tumor tissue to recognize and eliminate tumor cells [35]. Through this series of cellular responses, innate and adaptive immune responses are stimulated, resulting in a long-term immune response [36, 37].

Due to their good biodegradability and inherent immune effectiveness, nanoscale metal–organic frameworks (MOFs) with metal nodes and organic linkers can be used as multifunctional nanomaterials for immunostimulation-assisted therapy [38, 39]. MOFs are crystalline porous hybrids composed of metal ions or clusters with organic ligands. The introduction of porphyrins into MOFs as nano photosensitizers (PSs) improved the water solubility and pharmacokinetic properties of PSs, and also provided high loading and tumor-targeted delivery. The use of MOFs as sensitizers for photodynamic therapy (PDT) alleviated immunosuppressive TME and synergized with immunotherapeutic agents to stimulate antitumor-specific immunity.

**Scheme 1:**
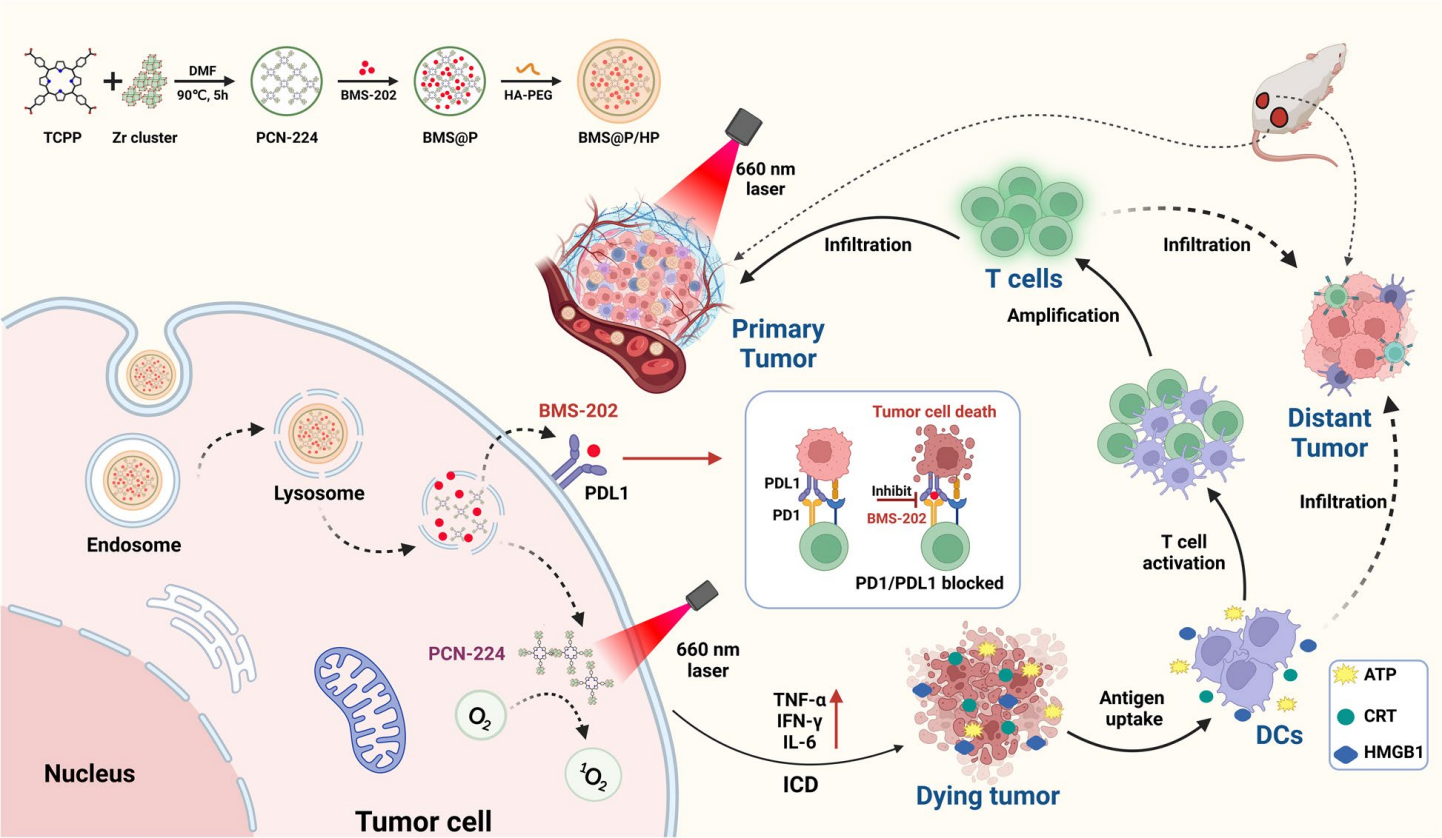
Diagram of photodynamic combined immunotherapy based on MOFs. Created with Biorender.com

In this study, we designed and constructed PCN-224 with a pore channel structure composed of Zr^4+^ and tetrakis (4-Carboxyphenyl) porphyrin (TCPP) using a solvothermal method, and then loaded PD-1/PD-L1 inhibitors BMS-202 in its pores via electrostatic adsorption, followed by surface modification with HA-PEG. The final product is called BMS@P/HP and is used to achieve a combination of photodynamic and immunotherapy as an integrated anti-tumor strategy, as shown in Scheme 1. Acid-responsive BMS@P/HP nanoparticles successfully deliver TCPP and BMS-202 simultaneously to cancer cells. Laser-irradiated TCPP induced massive ROS production, which significantly enhanced ICD. subsequently, DAMPs stimulated DCs maturation and generated further immune responses by loading BMS-202. The synergistic effect of PDT and immunotherapy upregulated the immunostimulatory signal, generating a cascade of immune responses that significantly improved the outcome of antitumor therapy (scheme 1).

## Materials and methods

### Materials

Hyaluronic acid (HA, 8000-10000 Da) were obtained from BLOOMAGE BIOTECHNOLOGY Co., Ltd. Methoxypolyethylene glycol amine (mPEG-NH_2_, M.W. 5000), Coumarin 6 (C-6, > 98%) and DCFH-DA (≥ 97%) were obtained from Aladdin Co., Ltd. N-Hydroxysuccinimide (NHS, 98%), EDC·HCl (> 97%), TCPP (> 98%), Zirconyl Chloride Octahydrate (ZrOCl_2_·8H_2_O, > 98%) and Benzoic Acid (99%) were obtained from Adamas. N, N-dimethylformamide (DMF, AR) and dimethyl sulfoxide (DMSO, AR) were obtained from China National Pharmaceutical Group Co., Ltd. BMS-202 were obtained from MedChemExpress. 1,3-Diphenylisobenzofuran (DPBF, > 97%) were obtained from TCI. Thiazolyl Blue Tetrazolium Bromide (MTT) were obtained from Solarbio.

### PCN-224 and BMS@P/HP Synthesis

We prepared PCN-224 by the solvothermal method [40]. 50 mg of TCPP, 150 mg of ZrOCl_2_·8H_2_O and 1.4 g of benzoic acid were dissolved in 70 ml of DMF, and the mixture was stirred at 90 °C for 5 h. After completion of the reaction, the product was collected by centrifugation (12 000 rpm, 30 min) and then washed once with fresh DMF and once with distilled water. PCN-224 was stored at 4 ℃ for subsequent experiments.

500 mg HA, 250 mg mPEG-NH_2_ and 460 mg NHS were dissolved in sodium tetraborate buffer at pH 8.5 and reacted for 30 min, followed by the addition of 1.2 g EDC and stirred at room temperature for 6 h. The reacted solution was transferred to a dialysis bag (MWCO = 7000 Da) and dialyzed in PBS at pH 7.4 for 24 h followed by dialysis in deionized water for 24 h. HA-PEG was obtained after lyophilization of the dialysate, and the structure of the material was determined by ^1^HNMR and FTIR.

200 μl of PCN-224 (5 mg/ml) and appropriate mass ratio of HA-PEG were added to 3 ml of distilled water, stirred for 30 min and centrifuged to obtain PCN-224/HP nanoparticles (PCN-224/HP NPs).

Add 200 μl of PCN-224 (5 mg/ml) and 80 μl of BMS-202 (5 mg/ml) to 3 ml of distilled water. The mixture was shaken overnight at 37 °C in a shaker. Then the appropriate mass ratio of HA-PEG was mixed with the above solution, stirred for 30 min, and centrifuged to obtain BMS@P/HP nanoparticles (BMS@P/HP NPs). The Co-Fc MOF Vk3 loading capability (LC) and encapsulation efficiency (EE) was measured as follows:

$$\mathrm{LC }\left(\mathrm{\%}\right)= \left(1-\frac{{A}_{ BMS-202\,in \,the \,supematant}}{{A}_{ BMS-202 \,before \,loading}}\right)\times \frac{{W}_{BMS-202}}{{W}_{BMS@P/HP}}\times 100\%$$

$$\mathrm{EE }\left(\mathrm{\%}\right)=\left(1-\frac{{A}_{ BMS-202 \,in\, the\, supematant}}{{A}_{ BMS-202\, before\, loading}}\right)\times 100\%$$

where A_BMS-202 in the supernatant_ corresponds to BMS-202 absorbance in supernatant samples, A_BMS-202 before loading_ corresponds to BMS-202 absorbance prior to loading. W_BMS-202_ corresponds to the initial mass of BMS-202, and W_BMS@P/HP_ corresponds to the BMS@P/HP mass.

### Nanoplatform characterization

UV–Vis absorption spectra were collected using a UV–Vis spectrophotometer (UT3C, YIPU). The morphology and elements of BMS@P/HP were analyzed by transmission electron microscopy (TEM, HT7800, HITACHI and Talos F200S, thermos Fisher). The crystal structure of the nanocarriers was characterized by x-ray diffraction (XRD, Ultima IV, Rigaku) and characterization of the nanocarriers by Fourier infrared spectroscopy (FTIR, Nicolet Is5, Thermo Fisher). The specific surface area and pore size of the nanoparticles were analyzed using a fully automated physisorption instrument (BET ASAP 2460, MICROMERITICS). Dynamic light scattering (DLS) was used to evaluate the hydrodynamic particle size and ζ-potential values with a Morven Zetazer Nano Series (Nano ZS90) instrument.

### Analyses of drug release

1 ml of PBS sample solution of BMS@P/HP (BMS-202, 400 μg/ml) was packed into several dialysis bags (MWCO = 3500 Da), which were then randomly placed in 11 ml of PBS buffer at pH 5.6, 6.5 and 7.4. Samples were constantly shaken at 100 rpm at 37 °C. At the set time points, 2 ml of dialysate was taken separately and supplemented with fresh PBS buffer to the original volume. The sample solution was centrifuged and the absorbance of the sample supernatant at 281 nm was measured by UV-Vis. Finally, the amount of BMS-202 released at different time points was calculated and the release curve was plotted.

### Extracellular singlet oxygen (^1^O_2_) Measurements

The extracellular photodynamic effect of PCN-224 was detected using DPBF as ^1^O_2_ trapping agent. The samples were set up in groups of DPBF, DPBF + H_2_O_2_, PCN-224 + DPBF + H_2_O_2_ (DPBF 10 mM), and the absorbance of DPBF was measured at 415 nm after irradiating the above solution with a 660 nm laser for different times.

2,2,6,6-tetramethylpiperidine (TEMP) was used as the ^1^O_2_ trapping agent, and the ability of different concentrations of PCN-224 to produce ^1^O_2_ was examined using electron spin resonance spectroscopy (ESR, A300, Bruker).

### Cell culture

Murine L929 fibroblasts were cultured in DMEM (BasalMedia) containing 10% FBS (NEM ZERUM) and penicillin/streptomycin (100x, BasalMedia) in a 37 °C 5% CO_2_ incubator. 4T1 breast cancer cells were cultured in RPMI-1640 (BasalMedia) containing 10% FBS (NEM ZERUM) and penicillin/streptomycin (100x, BasalMedia) in a 37 °C 5% CO_2_ incubator.

### Analyses of cell viability

L929 and 4T1 cells in the logarithmic growth phase were inoculated into 96-well plates (5 × 10^3^ cells/well) and cultured until the cells were plastered. A concentration gradient of BMS-202, PCN-224/HP NPs and BMS@P/HP NPs (diluted in sterile medium) was added and incubation was continued for 24 h. After 4 h incubation, PCN-224/HP NPs and BMS@P/HP NPs were irradiated with a 660 nm laser for 3 min per well, and then fresh medium containing drug was added and incubation was continued until 24 h. Cells without any treatment were set as the control group. The survival rate of cells was calculated using the MTT method.

### Live/Dead

The 4T1 cells were inoculated into 12-well plates (1 × 10^5^ cells/well) and incubated for 24 h, and then incubation was continued by adding medium containing BMS-202, PCN-224/HP NPs and BMS@P/HP NPs. The PCN-224/HP NPs and BMS@P/HP NPs groups were irradiated with a 660 nm laser for 3 min per well after 4 h of incubation, and then fresh medium containing the drug was added to continue incubation until 24 h. After 24 h, the cells were stained with Calcein-AM and propidium iodide (PI) (Calcein/PI Cell Activity and Cytotoxicity Assay Kit, Beyotime), respectively, and were observed by fluorescence microscopy and photographed.

### Analyses of intracellular ROS generation

Intracellular ROS production levels were measured with a DCFH-DA probe. 4T1 cells were inoculated on 24-well plates and cultured for 24 h. Media containing BMS-202, PCN-224/HP NPs and BMS@P/HP NPs were added and incubated for 4 h. Cells were irradiated with a 660 nm laser at 0.3 W/cm^2^ for 3 min in the dark. Then 20 μM DCFH-DA was immediately added, incubated for 30 min at 37 ℃, and detected by fluorescence microscopy (TH4-200, Olympus) and flow cytometry (FCM, Novo Cyte, Agilent).

### Apoptosis analyses

4T1 cells were inoculated on 12-well plates and cultured for 24 h. Media containing BMS-202, PCN-224/HP NPs and BMS@P/HP NPs were added and incubated for 4 h. Cells were irradiated with a 660 nm laser at 0.3 W/cm^2^ for 3 min in the dark. Cells were then stained using Annexin V/PI Apoptosis Detection Kit (Beyotime, C1062) for 15 min under light-proof conditions, and then was used for FCM analysis.

### Analyses of mitochondrial membrane potential

The 4T1 cells were inoculated on 24-well plates containing crawling tablets and cultured for 24 h. Media containing BMS-202, PCN-224/HP NPs and BMS@P/HP NPs were added and incubated for 4 h. Cells were irradiated with a 660 nm laser at 0.3 W/cm^2^ for 3 min in the dark. After a period of time, the cells were washed three times with PBS and stained with JC-1 (Mitochondrial membrane potential assay kit with JC-1, C2006, Beyotime) at 37 °C for 20 min. After fixed with 4% paraformaldehyde for 15 min, the nucleus was stained with DAPI, observed under a fluorescent microscope (Apo Tome 3, Zeiss) and photographed.

### Analyses of ICD induction

CRT exposure was detected by immunofluorescence staining. Briefly, the 4T1 cells were inoculated on 24-well plates containing crawling tablets and cultured for 24 h. Then, the samples were incubated with different treatments for 6 h. The samples were stained with anti-CRT (ET1608-60, HUABIO) and Alexa Fluor 594 coupling (HA1122, HUABIO) and detected by fluorescence imaging and FCM analysis. The same method was used for immunofluorescence detection of high mobility group protein (HMGB1, ER0913, HUABIO) release. Concentration gradient dilutions of ATP standards (10129ES03, Yeasen) were used for the preparation of ATP standard curves. the 4T1 cells were inoculated on 48-well plates and cultured for 24 h. Then, the samples were incubated with different treatments for 24 h. After 24 h, the supernatant was collected and the dead cells were removed by centrifugation. The ATP content in the supernatant was detected by ATP Luminescent cell viability assay kit (40210ES10, Yeasen). The relative luminescence units of the sample supernatant were quantified by multifunctional enzyme marker (CYT3MFV, Biotek).

### Tumor models

BALB/c mice (4-6 weeks old, female, SPF) from Chengdu Dashuo Experimental Animal Co., Ltd. were used to establish a subcutaneous 4T1 model of mouse breast cancer. Briefly, 1 × 10^6^ of 4T1 cells were injected subcutaneously into the right subcutis of each mouse. For the xenograft bilateral tumor model, 1 × 10^6^ of 4T1 cells were injected subcutaneously into the right subcutis of BALB/c mice on day -7, and 5 × 10^5^ 4T1 cells were injected subcutaneously into the left subcutis on day -3 (distant tumors). Treatment was performed when the tumor volume reached approximately 100 mm^3^.

### Combination PDT and PD-L1 blockade-based suppression of tumor growth

Subcutaneously tumor-bearing mice were randomly divided into 6 groups (*n* = 5) and injected tail vein with saline, PCN-224/HP NPs, BMS-202 and BMS@P/HP NPs (PCN-224 dose of 25 mg/kg and BMS-202 5 mg/kg), respectively. 4 h later, laser irradiation (660 nm, 0.3 W, 5 min) was performed on 2 groups of mice (PCN-224/HP NPs + L and BMS@P /HP NPs + L) were subjected to laser irradiation (660 nm, 0.3 W, 5 min). The drug was administered every two days for a total of three doses. The tumor size was measured every 2 days with calipers and the mice were weighed every two days, and the tumor volume was calculated as V _(mm_^3^_)_ = L × W^2^/2 (L is the maximum diameter and W is the minimum diameter).

### Exploring the mechanisms of in vivo combination therapy

On day 6 after the last treatment, mice were sacrificed and tumor tissues, blood and vital organs were collected. Tumor tissues from the subcutaneous tumor model were also used to analyze the tumor microenvironment. Detection of serum levels of IL-6, TNF-α and IFN-γ by ELISA. Tumors from mice were first excised and subsequently digested with collagenase IV (1 mg/ml) and DNase I (2u/ml) medium at 37 °C for 2 h. After 2 h, the tumor cell suspension was filtered through a 70 µm cell strainer to obtain single-cell suspensions. To analyze the proportion of tumor-infiltrating T cells, single cell suspensions were blocked by 10% goat serum for 30 min and used for further FCM analysis (L/D-PE-Texas Red/CD45-PCPcy5.5/CD3-APC/CD4-FITC/CD8a-PEcy7). Collect lymphocyte suspension by cutting open inguinal lymph nodes with a needle tip. To investigate the maturation of DCs, the obtained cell suspension was used for FCM analysis (CD11c-APC/CD80-FITC/CD86-PE). The spleen of mice was directly ground using a 70 µm cell strainer and lysed through red blood cell lysate to obtain single-cell suspensions of the spleen. To analyze the differentiation of T lymphocytes, single-cell suspensions from the spleen were used for FCM analysis (CD45-PCPcy5.5/CD3-APC/CD8-PEcy7/CD62L-FITC/CD44-PE).

### In vivo anti-tumor distal growth study of combination

Treatment was performed when the volume of the right subcutaneous tumor reached approximately 100 mm^3^. The treatment was performed as shown in 2.14. After the treatment, the mouse tissues were used for FCM analysis. The experimental method is shown in 2.15.

### Blood biochemical analysis

Blood was collected from the eyes of treated mice. The blood samples were left at room temperature for 10 h. The serum was then separated by centrifugation at 4000 r for 20 min at 4 °C. The mouse sera were assayed for relevant indexes by blood biochemical instruments.

### Tumor tissues analysis

After treatment, mice were dissected to collect organ and tumor tissues for histological analysis. To understand the mechanism of tumor necrosis, tumor sections were analyzed by H&E, TUNEL, Ki67 and CRT staining. CD4 and CD8 staining was performed on bilateral tumor sections to compare the immune cell infiltration of bilateral tumors. To assess the systemic toxicity of the agent, H&E staining was performed on major organs.

### Statistical analysis

The *P* value for data was calculated by two-way ANOVA. n.s. = non-significant, *P < 0.05, **P < 0.01, ***P < 0.005, ****P < 0.001.

## Result and discussion

### Synthesis and characterization of BMS@P/HP

**Fig. 1:**
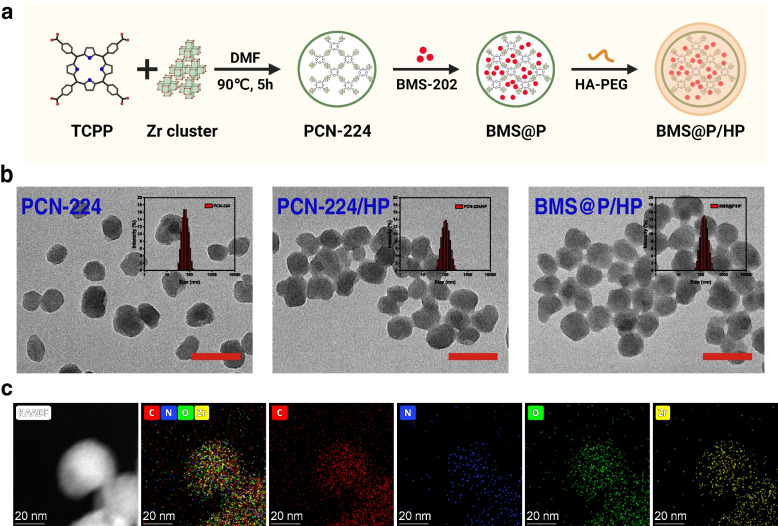
Synthesis and characterization of BMS@P/HP. **a** The scheme of synthetic procedure of the BMS@P/HP NPs. **b** TEM image of PCN-224, PCN-224/HP and BMS@P/HP NPs. Scale bar: 100nm. **c** Element mappings of BMS@P/HP NPs, including Zr, C, N and O. Scale bars: 20 nm

**Fig. 2:**
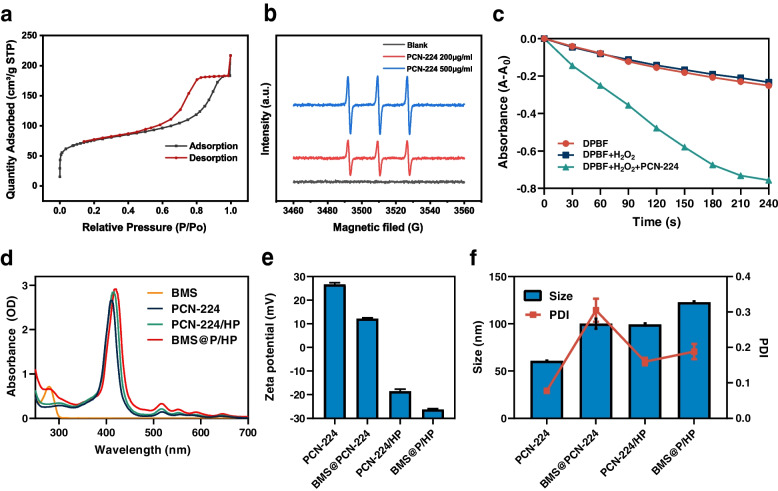
Characterization of BMS@P/HP. **a** Nitrogen adsorption–desorption isotherms and the corresponding pore size distribution of PCN-224 NPs. **b** ESR spectra were obtained after ^1^O_2_ capturing agent TEMP was added into blank, 200 μg/ml PCN-224 NPs and 500 μg/ml PCN-224 NPs aqueous solution irradiated by 660 nm laser for 5min. **c** Photodegradation rates of DPBF incubated with PCN-224 in the presence of H_2_O_2_ under 660 nm light irradiation. **d** UV-vis absorption spectra of BMS-202, PCN-224, PCN-224 /HP and BMS@P/HP. **e** ζ-potential and (**f**) hydrodynamic diameters of PCN-224, BMS@PCN-224, PCN-224 /HP and BMS@P/HP NPs

PCN-224 was prepared according to the method previously reported in the literature [41] (Fig. 1a). PCN-224 have a particle size of 60.8 nm (PDI = 0.078) and show a promising Tyndall effect in aqueous solution (Fig. 1b, Fig. S1a). TEM analysis of PCN-224 NPs shows that it is a spherical shape with a slightly uneven surface and a relatively uniform distribution (Fig. 1b). The crystals of PCN-224 have distinguishable diffraction peaks at 4.6, 6.48, 7.92, 9.14, 11.16, and 13.68 (Fig. S1c), proving the successful synthesis of PCN-224 NPs [42]. The existences of Zr, O, C and N in element mappings also demonstrate the successful preparation of PCN-224 (Fig. 1c). As seen from the UV–Vis absorption spectra (Fig. S1d), both TCPP and PCN-224 have characteristic absorption peaks at 419 nm and a series of weaker absorption bands in the visible region (500-650 nm). As seen on the FTIR spectrum of PCN-224 (Fig. S1e), the sample has asymmetric vibrational absorption at 1655 cm^−1^, due to the C = O group in TCPP. The broad signal located at 3435 cm^−1^ originates from O–H vibrations, indicating the presence of bonded and free water in all prepared samples. The vibrational bands near 1413 and 1601 cm^−1^ are characteristic of the -(O-C-O)- group, which confirms the presence of dicarboxylate in the product. The BET specific surface area of PCN-224 is 276.1510 m^2^/g and the average pore size is 6.91 nm (Fig. 2a). This porous structure makes PCN-224 to be a suitable nanocarrier.

We used ESR with TEMP as the trapping agent for the qualitative detection of ^1^O_2_ (Fig. 2b). Compared to the blank group, a typical 1:1:1:1 peak was observed in the PCN-224 spectrum after 5 min of 660 nm laser irradiation, and the signal intensity was further enhanced by increasing the sample concentration. It indicates that the ability of PCN-224 NPs to promote ^1^O_2_ production increases with increasing concentration. Meanwhile, we used DPBF as an indicator to verify the ability of PCN-224 to generate ^1^O_2_ (Fig. 2c). The absorbance of DPBF solution gradually decreased with the increase of light exposure time, which indicated that PCN-224 could promote the production of ^1^O_2_, further confirming the photosensitizer property of PCN-224.

The porous structure of PCN-224 allows it to be loaded with drugs. In order to improve stability, the surface of the BMS@PCN-224 is modified by HA-PEG (Fig. S2). UV-Vis absorption spectra confirmed that BMS-202 had been encapsulated in a BMS@P/HP sample (Fig. 2d) with a measured drug loading of 25.02%. DLS analyses indicated that the respective hydrodynamic diameters of BMS@PCN-224, PCN-224/HP and BMS@P/HP were 100.1 ± 5.3 nm (PDI = 0.31), 99.3 ± 1.1 nm (PDI = 0.16) and 122.8 ± 0.9 nm (PDI = 0.19) (Fig. 2f), respectively, with these values being larger than those measured via TEM as a consequence of their hydrated state (Fig. 1b, Fig S1b). Respective ζ-potential values for PCN-224, BMS@PCN-224, PCN-224/HP and BMS@P/HP were 26.6 ± 0.7, 12.1 ± 0.4, − 18.5 ± 0.9 and − 26.2 ± 0.4 mV (Fig. 2e).

BMS@P/HP exhibits rapid BMS-202 release rate in acidic medium (pH 5.5) (Fig. S2d). The cumulative release of BMS@P/HP NPs in different buffer solutions was overall lower than that of BMS@PCN-224 NPs, which was attributed to the fact that HA-PEG modification of the nanoparticles' surfaces slowed down the release rate and reduced the abrupt release, which was favorable for the long-term function of the nanoparticles in vivo.

### Evaluation of biological performances of nanocomplexes in Vitro

To investigate the uptake behavior of the nanocomplexes, we used C-6 as a fluorescent marker for PCN-224/HP NPs and examined its uptake. C-6 was able to be excited by laser at 488 nm and detected in the FITC channel. The number of green particles in the cells increases with the prolongation of incubation time and the fluorescence intensity of the cells increases with the prolongation of time (Fig. S3a, b), as seen on the FCM assay. The two experimental results are consistent, indicating that the nanoparticles are taken up by the tumor cells according to the time dependence. After 2 h of incubation, the green signal of the nanocomposite overlapped with the red signal of the lysosomes (in yellow color), and an enhanced yellow signal was found in the cells after 4 h. When the incubation time was extended, some nanoreactors escaped from the lysosomes (Fig. S3c).

**Fig. 3:**
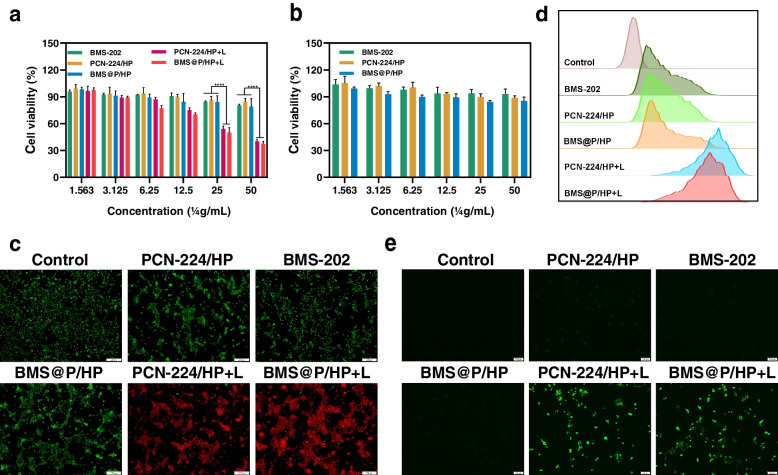
In vitro antitumor effects of nanocomplexes. **a** The cell cytotoxicity of BMS-202, PCN-224/HP and BMS@P/HP NPs for the normal cell L929 by MTT assay. **b** The cell cytotoxicity of BMS-202, PCN-224/HP and BMS@P/HP NPs for the tumor cell 4T1 by MTT assay. (*n* = 3, *P < 0.05, **P < 0.01, ***P < 0.001, ***P < 0.001, ****P < 0.0001). **c** After treatment with different drugs, 4T1 cells were tested for LIVE/DEAD. Living and dead cells were stained with Calcein-AM (green) and PI (red), respectively. Scale bar: 100 μm. **d** Flow cytometry and (**e**) fluorescence microscopy were used to detect ROS levels in 4T1 cells. Scale bar: 100 μm

The cytotoxicity of nanocomposite against cancer cells was evaluated. Notably, the cell viability of L929 cells remained almost unchanged after treatment with PCN-224/HP and BMS@P/HP in the concentration range of 1.56-50.00 μg/ml of PCN-224 (Fig. 3a), indicating their good cytocompatibility. Theoretically, BMS-202 is a non-peptide PD-1/PD-L1 complex inhibitor that blocks the PD-L1/PD-1 signaling pathway, but has no tumor-killing effect on tumor cells [43, 44]. The cell viability obviously decreased after the cells were treated with nanocomplexes and 660 nm laser irradiation (Fig. 3b). Calcein-AM does not fluoresce and is hydrolyzed by endogenous esterases in living cells upon entry to generate Calcein, which is impermeable to cell membranes, emitting strong green fluorescence. The cell-impermeable PI can only stain dead cells whose cell membrane integrity has been disrupted. Compared to the unirradiated group, the nanocomposite group that received 660 nm laser irradiation showed a stronger red fluorescent signal, indicating that the 4T1 cells were dead (Fig. 3c), which was consistent with the MTT results. In addition, we also confirmed that photodynamic therapy can inhibit the proliferation and migration of 4T1 cells in vitro by clonal cluster assay (Fig. S3d).

ROS are a normal by-product of many cellular metabolisms. High levels of ROS cause damage to proteins, nucleic acids, lipids, cell membranes and organelles, leading to cell death [45]. We used DCFH-DA as a ROS probe to assay the ability of nanocomplexes receiving 660 nm laser irradiation to generate ROS in 4T1 cells. The non-fluorescent DCFH-DA was hydrolyzed by intracellular esterases to generate DCFH, which could not cross the cell membrane thus allowing it to be easily loaded into the cell. DCFH could be oxidized to green fluorescent DCF by intracellular ROS. The nanocomposite group that received 660 nm laser irradiation showed intense green fluorescence compared to the control group (Fig. 3e), indicating that PCN-224 self-delivery nanocarriers can be internalized by cells and exert PDT effects under 660 nm laser irradiation. As seen in the FCM assay (Fig. 3d), the PCN-224/HP NPs, BMS-202 and BMS@P/HP NPs groups had very faint green fluorescence compared to the control group, which may be due to the effect of the added drug on cellular metabolism.

**Fig. 4:**
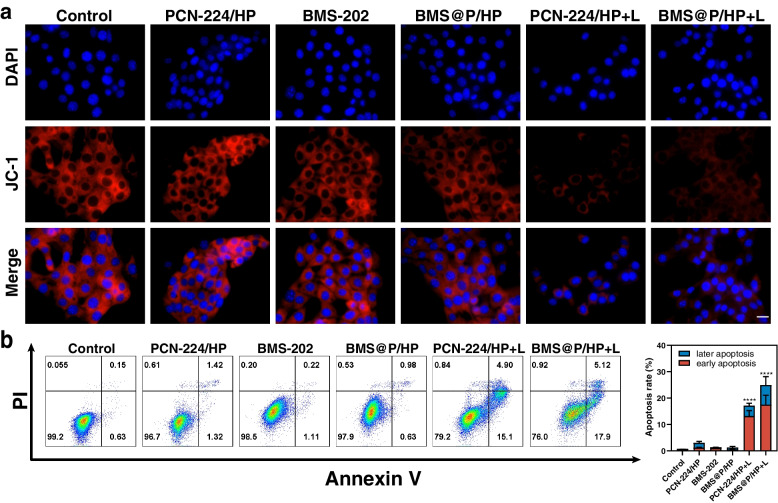
PDT leading to apoptotic cell death in 4T1 cells. **a** The mitochondrial membrane potential of 4T1 cells was detected by JC-1. Scale bar: 20 μm. **b** Annexin V-FITC/PI apoptosis kit was used to detect the apoptosis of 4T1 cells and analyze the quantitative apoptosis rate. (*n* = 3, *P < 0.05, **P < 0.01, ***P < 0.001, ***P < 0.001, ****P < 0.0001)

Mitochondria play an important role in initiating apoptosis, and high levels of mitochondrial ROS initiate intrinsic apoptosis [45, 46]. JC-1 is an ideal fluorescent probe widely used to detect the mitochondrial membrane potential ∆Ψm. At high mitochondrial membrane potential, JC-1 aggregates in the mitochondrial matrix to form polymers (J-aggregates) and produce red fluorescence. The decrease in mitochondrial membrane potential is a landmark event in the early stages of apoptosis. According to the analysis of treated 4T1 cells, the red fluorescence of the nanocomposite group receiving irradiation became weaker, indicating that the mitochondrial membrane potential decreased and 4T1 cells experienced early apoptosis (Fig. 4a). Next, we analyzed the Annexin V-FITC/PI-stained treated 4T1 cells by flow cytometry (Fig. 4b). Compared with the blank control group, the PCN-224/HP NPs, BMS-202, BMS@P/HP NPs, PCN-224/HP + L and BMS@P/HP + L groups all showed different degrees of tumor cell apoptosis, and they induced apoptosis rates of 3.04 ± 0.56, 1.33 ± 0.08, 1.27 ± 0.62, respectively. This further confirmed that PDT induced disruption of mitochondrial membrane potential leading to apoptosis of tumor cells.

**Fig. 5:**
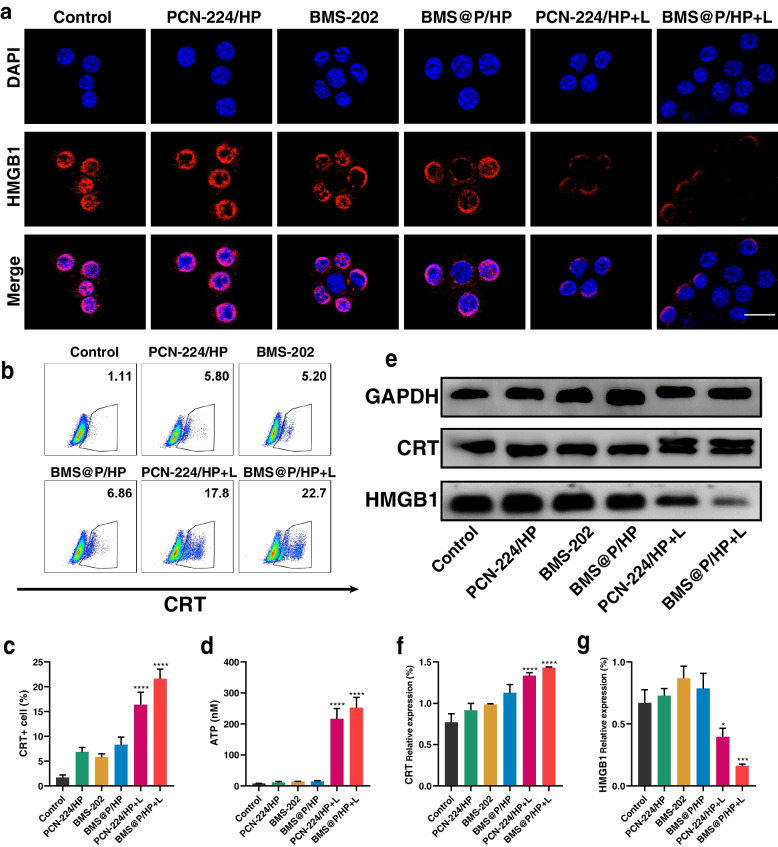
PDT induced ICD in vitro. **a** HMGB1 immunofluorescence staining of 4T1 cells in different treatment groups. Scale bar: 20 μm. **b**, **c** Detection of cell surface CRT expression using FCM. **d** Extracellular ATP secretion in 4T1 cells treated with different drugs. **e**, **f**, **g** Expression levels of CRT and HMGB1 were analyzed by Western blot. (*n* = 3, *P < 0.05, **P < 0.01, ***P < 0.001, ***P < 0.001, ****P < 0.0001)

PDT has been shown to induce ICD and elicit an antitumor immune response due to its strong immunogenicity. Tumor cells undergoing ICD transport CRT to the cell surface and release HMGB1 and ATP. this promotes phagocytosis of dead tumor cells and their fragments by antigen-presenting cells. For the purpose of investigating the ICD effect of PCN-224 NPs activation, we examined critical ICD biomarkers. 4T1 cells treated with nanocomplexes and laser irradiation emitted strong red fluorescence by immunofluorescence analysis (Fig. S3e), indicating enhanced exposure of CRT on the cell surface. Besides, according to FCM analysis (Fig. 5b, c), PDT could significantly increase the expression of CRT on the surface of 4T1 cells. From the analysis of immunofluorescence data (Fig. 5a), it is clear that laser-irradiated and nanocomposite-treated 4T1 cells exhibit a very faint red fluorescence compared to the unirradiated cells, a result that indicates efficient extracellular release of HMGB1. To further assess the expression of CRT and HMGB1, we performed Western blot analysis and quantification of 4T1 cells after different treatments (Fig. 5e, f, g). CRT expression was significantly increased and HMGB1 expression was decreased in 4T1 cells treated with laser irradiation and nanocomplexes, confirming a strong ICD response in tumor cells. In addition, extracellular ATP secretion was significantly higher in 4T1 cells treated with laser irradiation and nanocomplexes than in the other groups (Fig. 5d), which was attributed to ROS-induced mitochondrial damage leading to apoptosis of tumor cells, a process accompanied by ATP secretion. In overview, self-delivered nano produced apoptosis-inducing ROS under laser irradiation and had a good ICD effect.

### Antitumor immune responses of combination therapy in single 4T1 tumor-Bearing mice model

The biodistribution of free TCPP and PCN-224/HP NPs in 4T1 tumor-bearing mice was assessed by animal live imager (Fig. S4a). The fluorescence signal of the free TCPP gradually diminished over time at the tumor site, but gradually increased at the liver site. In contrast, PCN-224/HP NPs had a significant fluorescence signal at the tumor site and were retained longer than the free TCPP. These results suggested that the nanocomplexes are able to remain at the tumor site for a long time. As seen in the tumor frozen section that the Cy5.5@P/HP NPs group has more distribution in the tumors, which indicated that MOFs-nanocarriers are beneficial for targeting tumors (Fig S4b, S4c).

**Fig. 6:**
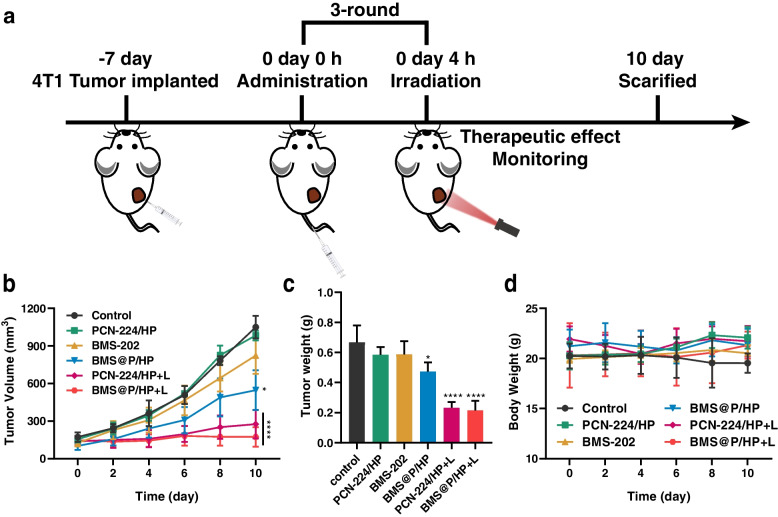
In vivo antitumor effect of PDT combined with iPD-L1. **a** Establishment and treatment of 4T1 subcutaneous tumor model. **b** Tumor growth curve, **c** tumor weight and **d** body weight in mice

Subsequently, we evaluated the therapeutic effect of BMS@P/HP on 4T1 tumor-bearing BALB/c mice according to the treatment protocol (Fig. 6a). There was no significant anti-tumor effect of PCN-224/HP compared to control. Free BMS-202 and BMS@P/HP showed weak antitumor efficacy during administration, but the inhibition of tumor growth was not significant after stopping the administration. The nanocomplexes subjected to 660 nm laser irradiation all showed significant tumor-suppressive effects, and the effect of BMS@P/HP + L was more pronounced than that of PCN-224/HP + L. The treated tumor volume (Fig. 6b) and weight (Fig. 6c) showed consistent anti-tumor effects. This suggests a significant antitumor effect of nanocomplexes-mediated PDT, a process that may promote antitumor immunity by inducing ICD and subsequent TAA release, thereby clearing the way for immune checkpoint inhibitors to work. Thus, the tumor suppressive effect of the combination therapy was significantly stronger than that of the monotherapy.

**Fig. 7:**
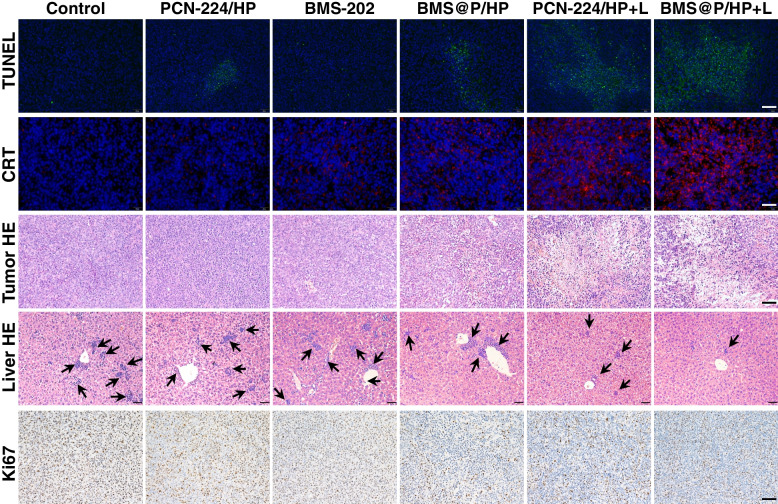
TUNEL, CRT, H&E and Ki67 histochemical sections of mouse tumors and H&E histochemical sections of liver in different treatment groups. Scale bar: 50 μm

At the end of treatment, tumor sections from mice were analyzed by TUNEL, CRT, Ki67 and H&E to further assess the anti-tumor effect of the combined strategy (Fig. 7). Cell shrinkage in the nanocomplexes group receiving 660 nm laser irradiation was observed in the H&E-stained tumor tissue sections, which differed from the intact cell morphology of the untreated group. Ki67 and TUNEL staining of tumor tissue sections showed that the PCN-224/HP + L and BMS@P/HP + L groups induced more tumor necrosis and apoptosis compared to the other treated groups. In H&E-stained liver tissue sections, black arrows point to tumor metastases in the liver. It could be seen that the tumors in the PCN-224/HP + L and BMS@P/HP + L groups had fewer metastasis in the liver. Also, the experimental results confirmed that there was no significant liver toxicity at the end of the treatment. Such results indicated that our MOFs are highly efficient and low toxicity. We performed CRT immunofluorescence staining on the tumor sections. We also found that tumors in the PCN-224/HP + L and BMS@P/HP + L groups had fewer metastases in the liver sites. The results showed that both PCN-224/HP + L and BMS@P/HP + L groups showed significant red fluorescence, indicating that PDT significantly induced the ICD effect. Besides, the fluorescence of the BMS@P/HP + L group was significantly stronger than that of PCN-224/HP + L, which might be because iPD-L1 alleviated the immunosuppressive microenvironment favoring the induction of ICD. These results suggested that the combination strategy achieved significant antitumor effects.

**Fig. 8:**
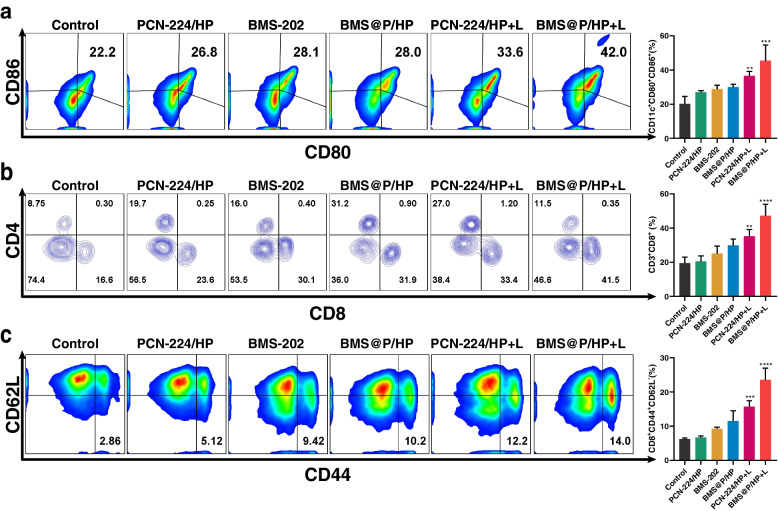
Exploration of immune mechanisms in a single 4T1 subcutaneous tumor. **a** The proportion of mature DCs (CD80 + CD86 +) in lymph nodes near the tumor was analyzed by FCM. **b** FCM analysis of the proportions of CD4 + and CD8 + to CD3 + T cells in tumors. **c** The proportion of TEM (CD8 + CD44 + CD62L-) in spleen. (*n* = 3, *P < 0.05, **P < 0.01, ***P < 0.001, ***P < 0.001, ****P < 0.0001)

We further investigated the immune response induced by PDT in combination with iPD-L1. It is well known that DCs play an irreplaceable role in the adaptive immune process. ICD induces the accumulation and maturation of DCs, which further stimulates T-cell maturation and initiates the immune cycle to achieve tumor killing. The cell surface of mature DCs expresses co-stimulatory molecules (CD80/CD86), which are required to activate the T-cell response. For this purpose, we examined the proportion of mature DCs in the lymph nodes near the tumor by FCM (Fig. 8a). The proportion of mature DCs in the PCN-224/HP + L and BMS@P/HP + L treated groups was 1.5 and 1.9 times higher than that in the control group, respectively.

T cells are critical for tumor surveillance. In order to generate a durable anti-tumor immune response, activation and infiltration of T cells into the tumor region is essential. Therefore, we detected the proportion of cytotoxic T lymphocytes (CTLs, CD3^+^CD8^+^) infiltrating the tumor site by FCM (Fig. 8b). The proportions of CTLs in PCN-224/HP + L and BMS@P/HP + L were 2 and 2.5 times higher than those in the control, respectively. These results indicated that the ICD effect stimulated the maturation of DCs and further promoted the proportion of infiltrating CTLs in the tumor, verifying the activation of the immune response in immunotherapy. It was also evident from the above data that PDT combined with iPD-L1 was more effective due to the fact that BMS-202 blocked the binding of PD-1 on the surface of T cells to PD-L1 ligands of tumor cells making T cells easier to be activated in the tumor microenvironment.

To further investigate the long-term immunological memory effect induced by the combination treatment, we examined the proportion of effector memory T cells (T_EM_, CD8^+^CD44^+^CD62L^−^) in the spleen of mice. The proportion of effector T cells and memory T cells was highest in mice treated with BMS@P/HP + L compared with other groups (Fig. 8c), suggesting a significant effect of immune memory induced by combination treatment. Cytokines are involved in almost all types of cellular responses, such as regulation of immune proliferation, differentiation and effector functions, and are essential for immune cells against tumor cells and pathogens [47]. We examined three key immune-related cytokines, TNF-α, IFN- γ and IL-6 (Fig. S4d, e, f). We found that the laser-irradiated nanocomposite-treated group had higher cytokine levels than the other groups.

### Combination therapy against distant tumor growth investigation

**Fig. 9:**
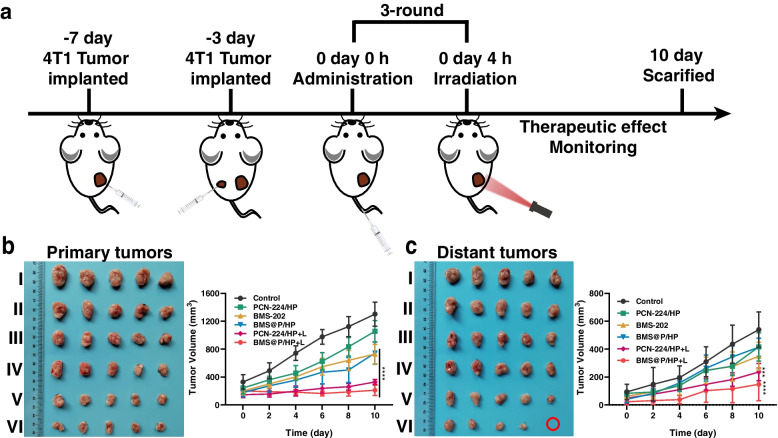
PDT combined with iPD-L1 against distal tumor development. **a** Establishment and treatment of bilateral 4T1 tumor model. (**b**) Primary tumors and (**c**) distant tumors images and tumor growth curve of different treatment groups

We established a xenograft bilateral 4T1 tumor model to investigate the effect of combination therapy against distal tumor growth, administered according to the previous treatment regimen (Fig. 9a). Similar to the treatment results in a single 4T1 tumor-bearing mouse model, primary tumor proliferation was inhibited in the nanocomplexes receiving 660 nm laser irradiation (Fig. 9b, S4g, S4h). The distant tumor growth was significantly inhibited in the BMS@P/HP + L group due to upregulation of the body's immune function (Fig. 9c, S4i).

**Fig. 10:**
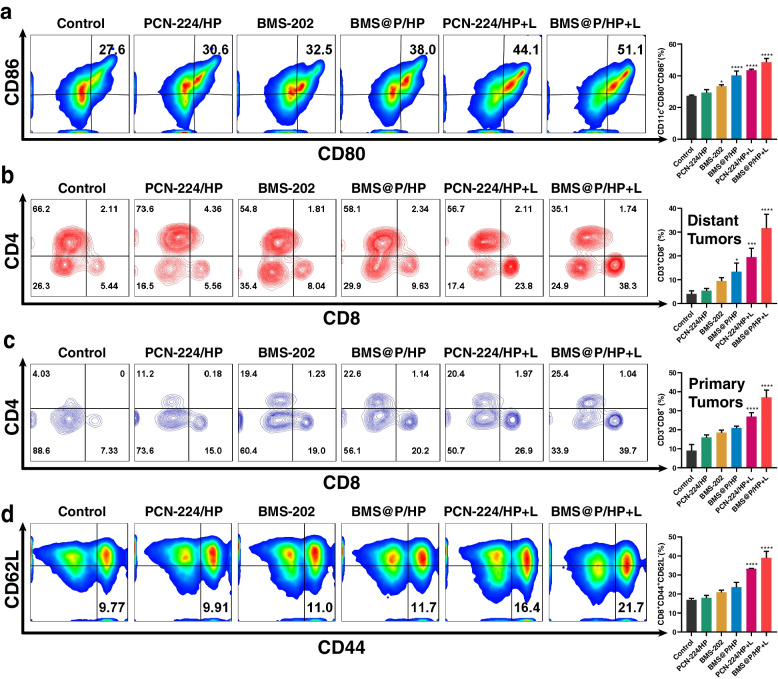
Exploration of immune mechanisms bilateral 4T1 tumor model. **a** The proportion of mature DCs (CD80 + CD86 +) in lymph nodes near the tumor was analyzed by flow cytometry. **b** Flow cytometry analysis of the proportions of CD4 + and CD8 + to CD3 + T cells in the distant and primary tumors. **c** The proportion of TEM (CD8 + CD44 + CD62L-) in spleen. (*n* = 3, *P < 0.05, **P < 0.01, ***P < 0.001, ***P < 0.001, ****P < 0.0001)

Further validation of the tumor immune response was performed. The proportion of mature DCs in the lymph nodes of mice treated with nanocomplexes and laser irradiation increased (Fig. 10a), as did the proportion of CTLs infiltrated by tumors on both sides (Fig. 10b). This result confirmed the upregulation of the immune level in the mouse organism. We performed immunofluorescence staining of bilateral tumor sections to examine the infiltration of T cells in primary and distant tumors (Fig. S5a). CD4/CD8 fluorescence in the PCN-224/HP group was faintly similar to that in the control group. The number of CTLs was increased in both primary and distant tumors in the PCN-224/HP + L and BMS@P/HP + L groups compared with the control group. T cell infiltration of tumors was greatest in the BMS@P/HP + L group, which may be due to the effect of iPD-L1. The proportion of T_EM_ in the spleen of mice (Fig. 10c) was also similar to the results of a single 4T1 tumor model, suggesting the development of a long-term immune memory effect, which favors the suppression of distal tumor development.

Importantly, the treated mice showed little change in body weight (Fig. S4j), similar to the control group. Similarly, H&E images of sections of major organs (heart, spleen, lung and kidney) of mice showed the biological safety of the nanocomplexes (Fig. S5b). The results of blood biochemical assays are shown that the absence of significant changes in vital parameters in the treated group indicates negligible hepatotoxicity of BMS@P/HP (Fig. S5c).

## Discussion

Combination therapeutic strategies based on MOFs should be more careful in the selection of treatment methods. First, a better toxicity evaluation system and drug metabolism assessment system should be established to design a reasonable dose and frequency of administration to reduce the damage of MOFs to the organism; then, the detailed mechanism of MOFs involved in immunotherapy needs to be further explored; finally, the excessive immunity of combination therapies in vivo should be carefully considered, and it is necessary to design a rational synergistic treatment system based on the understanding of the exact mechanism of different treatments.

## Conclusion

In summary, we reported a novel BMS@P/HP nanocomplexes that achieved a synergistic anti-tumor strategy. Nanocomplexes that received 660 nm laser irradiation converted intracellular oxygen molecules into ROS, which promoted tumor cell necrosis and apoptosis. PCN-224-mediated PDT enhanced ICD, which promoted DCs maturation and further intratumoral infiltration of CTLs. Nanocomplexes-carrying BMS-202 blocked PD1 binding to PD-L1 leading to TME remodeling. Thus, BMS@P/HP + L could enhance primary and distant specific anti-tumor T cell responses and inhibit tumor progression in a bilateral 4T1 model. This study provides a promising platform for MOF-mediated PDT combined with iPD-L1 anti-tumor strategy.

### Supplementary Information

**Additional file 1: Fig. S1.** (**a**) The picture of (i) PCN-224, (ii)PNC-224/HP and (iii) BMS@P/HP NPs. (**b**) TEM image of BMS@PCN-224 NPs. Scale bar: 100nm. (**c**) Powder X-ray diffraction spectra of PCN-224 NPs. (**d**) The UV spectrum of TCPP and PCN-224 NPs. (**e**) FTIR spectrums of PCN-224 NPs. **Fig. S2. **(**a**)Synthesis route of HA-PEG. (**b**) The ^1^H-NMR spectrum of HA, mPEG-NH_2_ and HA-PEG. (**c**) The FTIR spectrum of HA, mPEG-NH2 and HA-PEG. In vitro release profiles of BMS, BMS@PCN-224 and BMS@P/HP at pH (**d**) 5.5, (**e**) 6.5 and (**f**) 7.4. **Fig. S3.** The uptake of C-6@PCN-224/HP NPs in 4T1 cells was determined by (**a**) fluorescence microscopy and (**b**) FCM. (**c**) Study on colocalization of C-6@PCN-224/HP NPs and lysosomes. Scale bar: 20 μm. (**d**) Experiments on cell clone clusters treated with different drugs. (**e**) CRT immunofluorescence staining of 4T1 cells in different treatment groups. Scale bar: 20 μm. **Fig. S4.** (**a**) Biodistribution of Cy5.5 and Cy5.5@P/HP NPs in 4T1 tumor bearing mice. Frozen section of tumors in (**b**) Cy5.5 and (**c**) Cy5.5@P/HP NPs groups. Tumor cells were stained with blue and the cy5.5 is red. Scale bar: 50 μm. Serum levels of cytokines (**d**) IFN-γ, (**e**) IL-6 and (**f**) TNF-α in different treatment groups. (**g**) Distant tumor and (**h**) primary tumor weight of mice. (**i**) Body weight in bilateral 4T1 tumor model mice. **Fig. S5.** (**a**) CD4 and CD8 immunofluorescence staining of distant and primary tumor sections. Scale bar: 50 μm. (**b**) H&E staining of the major organs. Scale bar: 100 μm. (**c**) Serum biochemical analysis of mice in each treatment group.

## Data Availability

Not applicable, please refer to the original references.

## Abbreviations

TNBC: Triple-negative breast cancer
PDT: Photodynamic therapy
PSs: Photosensitizers
ROS: Reactive oxygen species
CRT: Calreticulin
ICD: Immunogenic cell death
DAMPs: Danger associated molecular patterns
MOFs: Metal-organic frameworks
TCPP: Tetrakis (4-Carboxyphenyl) Porphyrin
DCs: Dendritic cells
HA: Hyaluronic acid
PEG: Polyethylene glycol
DLS: Dynamic light scattering
TEM: Transmission Electron Microscope
FCM: Flow cytometry
TME: Tumor microenvironment
HMGB1: High mobility group protein
MTT: Thiazolyl Blue Tetrazolium Bromide
CTLs: Cytotoxic T lymphocytes
