# Supplementary figures and images for: Metal-organic framework-based photodynamic combined immunotherapy against the distant development of triple-negative breast cancer
Source: Biomater Res. 2023 Nov 24;27:120. doi: 10.1186/s40824-023-00447-x (PMC10668380; doi:10.1186/s40824-023-00447-x)

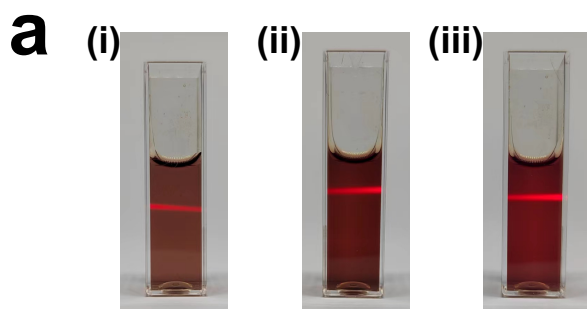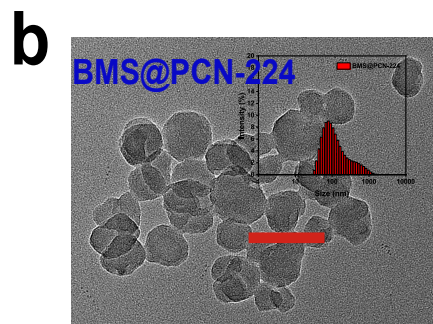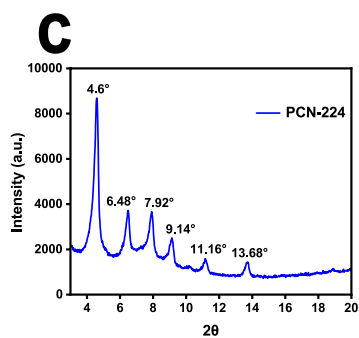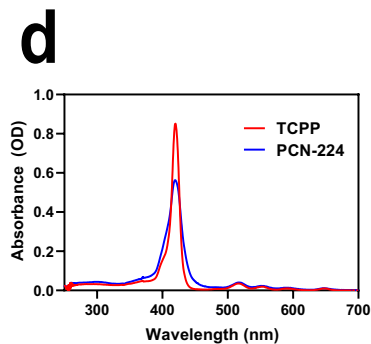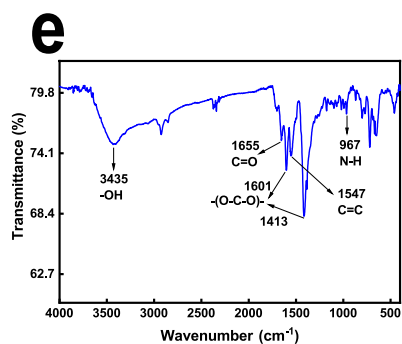

Supplement: Supplementary file 1 — Additional file 1: Fig. S1. (a) The picture of (i) PCN-224, (ii)PNC-224/HP and (iii) BMS@P/HP NPs. (b) TEM image of BMS@PCN-224 NPs. Scale bar: 100nm. (c) Powder X-ray diffraction spectra of PCN-224 NPs. (d) The UV spectrum of TCPP and PCN-224 NPs. (e) FTIR spectrums of PCN-224 NPs. Fig. S2. (a)Synthesis route of HA-PEG. (b) The 1H-NMR spectrum of HA, mPEG-NH2 and HA-PEG. (c) The FTIR spectrum of HA, mPEG-NH2 and HA-PEG. In vitro release profiles of BMS, BMS@PCN-224 and BMS@P/HP at pH (d) 5.5, (e) 6.5 and (f) 7.4. Fig. S3. The uptake of C-6@PCN-224/HP NPs in 4T1 cells was determined by (a) fluorescence microscopy and (b) FCM. (c) Study on colocalization of C-6@PCN-224/HP NPs and lysosomes. Scale bar: 20 μm. (d) Experiments on cell clone clusters treated with different drugs. (e) CRT immunofluorescence staining of 4T1 cells in different treatment groups. Scale bar: 20 μm. Fig. S4. (a) Biodistribution of Cy5.5 and Cy5.5@P/HP NPs in 4T1 tumor bearing mice. Frozen section of tumors in (b) Cy5.5 and (c) Cy5.5@P/HP NPs groups. Tumor cells were stained with blue and the cy5.5 is red. Scale bar: 50 μm. Serum levels of cytokines (d) IFN-γ, (e) IL-6 and (f) TNF-α in different treatment groups. (g) Distant tumor and (h) primary tumor weight of mice. (i) Body weight in bilateral 4T1 tumor model mice. Fig. S5. (a) CD4 and CD8 immunofluorescence staining of distant and primary tumor sections. Scale bar: 50 μm. (b) H&E staining of the major organs. Scale bar: 100 μm. (c) Serum biochemical analysis of mice in each treatment group. [file 40824_2023_447_MOESM1_ESM.zip › S1_ESM.pdf]

**a**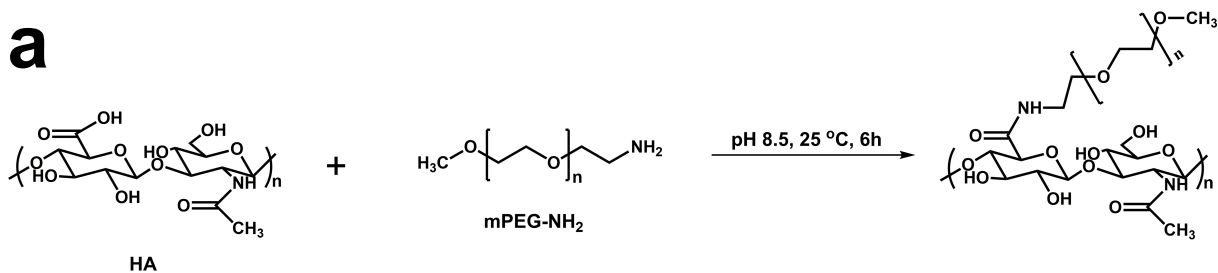**b**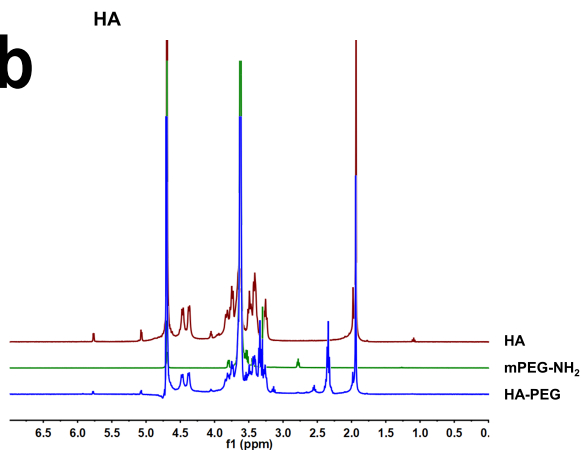**c**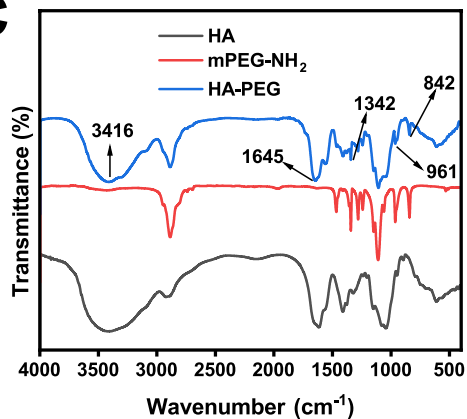**d**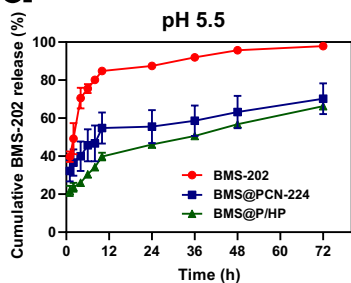**e**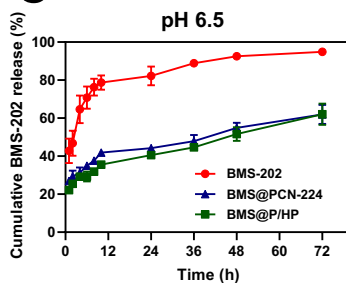**f**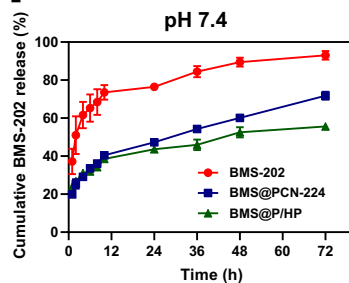

Supplement: Supplementary file 1 — Additional file 1: Fig. S1. (a) The picture of (i) PCN-224, (ii)PNC-224/HP and (iii) BMS@P/HP NPs. (b) TEM image of BMS@PCN-224 NPs. Scale bar: 100nm. (c) Powder X-ray diffraction spectra of PCN-224 NPs. (d) The UV spectrum of TCPP and PCN-224 NPs. (e) FTIR spectrums of PCN-224 NPs. Fig. S2. (a)Synthesis route of HA-PEG. (b) The 1H-NMR spectrum of HA, mPEG-NH2 and HA-PEG. (c) The FTIR spectrum of HA, mPEG-NH2 and HA-PEG. In vitro release profiles of BMS, BMS@PCN-224 and BMS@P/HP at pH (d) 5.5, (e) 6.5 and (f) 7.4. Fig. S3. The uptake of C-6@PCN-224/HP NPs in 4T1 cells was determined by (a) fluorescence microscopy and (b) FCM. (c) Study on colocalization of C-6@PCN-224/HP NPs and lysosomes. Scale bar: 20 μm. (d) Experiments on cell clone clusters treated with different drugs. (e) CRT immunofluorescence staining of 4T1 cells in different treatment groups. Scale bar: 20 μm. Fig. S4. (a) Biodistribution of Cy5.5 and Cy5.5@P/HP NPs in 4T1 tumor bearing mice. Frozen section of tumors in (b) Cy5.5 and (c) Cy5.5@P/HP NPs groups. Tumor cells were stained with blue and the cy5.5 is red. Scale bar: 50 μm. Serum levels of cytokines (d) IFN-γ, (e) IL-6 and (f) TNF-α in different treatment groups. (g) Distant tumor and (h) primary tumor weight of mice. (i) Body weight in bilateral 4T1 tumor model mice. Fig. S5. (a) CD4 and CD8 immunofluorescence staining of distant and primary tumor sections. Scale bar: 50 μm. (b) H&E staining of the major organs. Scale bar: 100 μm. (c) Serum biochemical analysis of mice in each treatment group. [file 40824_2023_447_MOESM1_ESM.zip › S2_ESM.pdf]

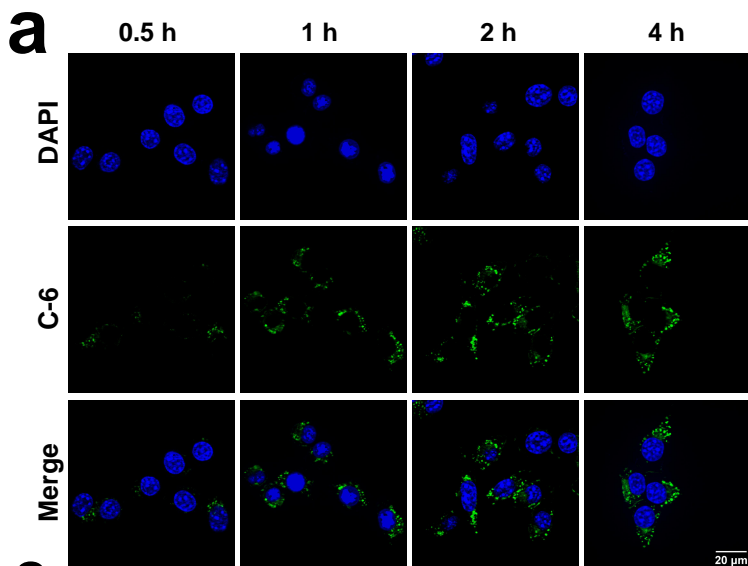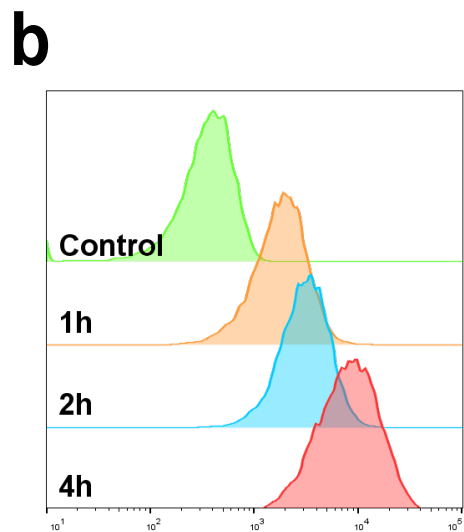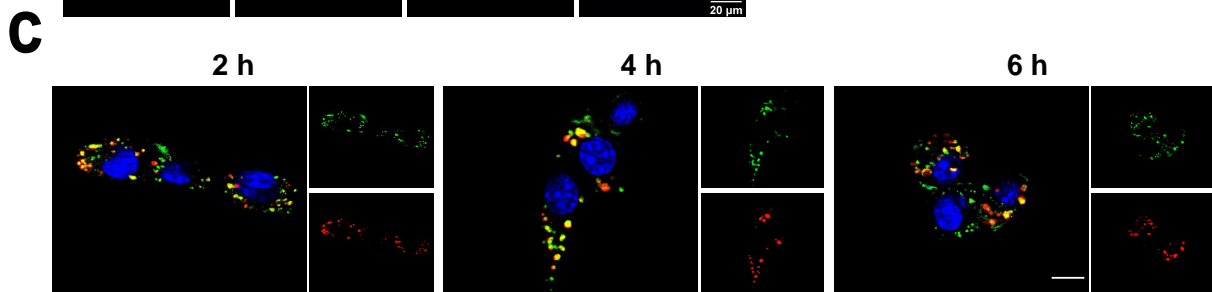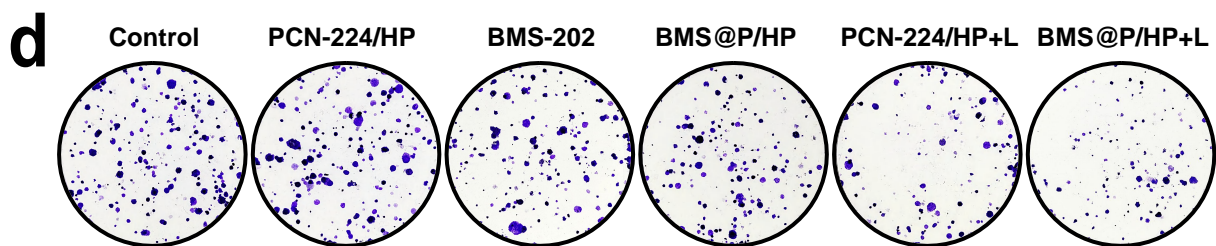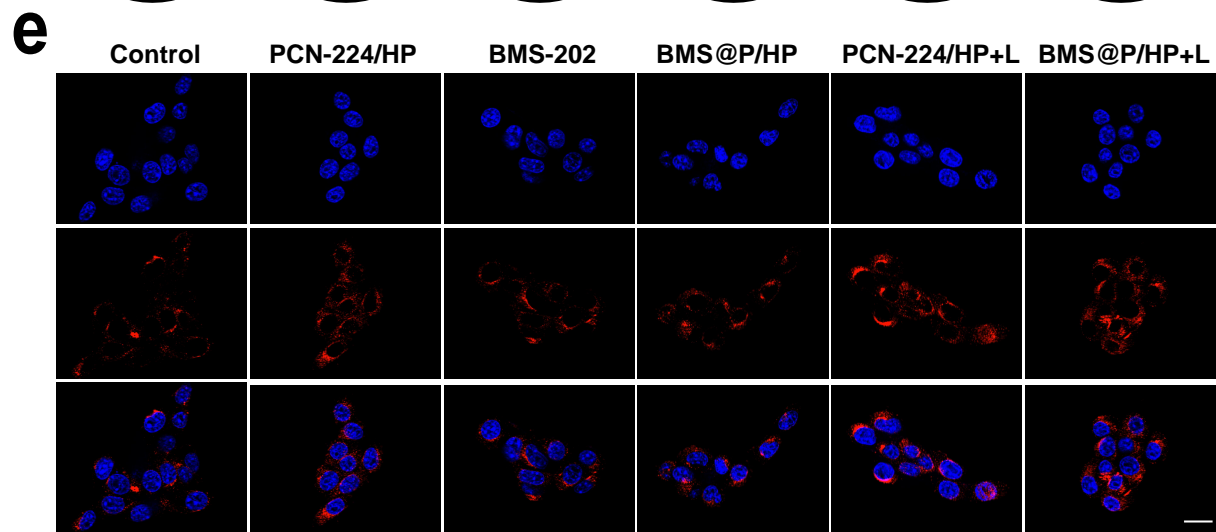

Supplement: Supplementary file 1 — Additional file 1: Fig. S1. (a) The picture of (i) PCN-224, (ii)PNC-224/HP and (iii) BMS@P/HP NPs. (b) TEM image of BMS@PCN-224 NPs. Scale bar: 100nm. (c) Powder X-ray diffraction spectra of PCN-224 NPs. (d) The UV spectrum of TCPP and PCN-224 NPs. (e) FTIR spectrums of PCN-224 NPs. Fig. S2. (a)Synthesis route of HA-PEG. (b) The 1H-NMR spectrum of HA, mPEG-NH2 and HA-PEG. (c) The FTIR spectrum of HA, mPEG-NH2 and HA-PEG. In vitro release profiles of BMS, BMS@PCN-224 and BMS@P/HP at pH (d) 5.5, (e) 6.5 and (f) 7.4. Fig. S3. The uptake of C-6@PCN-224/HP NPs in 4T1 cells was determined by (a) fluorescence microscopy and (b) FCM. (c) Study on colocalization of C-6@PCN-224/HP NPs and lysosomes. Scale bar: 20 μm. (d) Experiments on cell clone clusters treated with different drugs. (e) CRT immunofluorescence staining of 4T1 cells in different treatment groups. Scale bar: 20 μm. Fig. S4. (a) Biodistribution of Cy5.5 and Cy5.5@P/HP NPs in 4T1 tumor bearing mice. Frozen section of tumors in (b) Cy5.5 and (c) Cy5.5@P/HP NPs groups. Tumor cells were stained with blue and the cy5.5 is red. Scale bar: 50 μm. Serum levels of cytokines (d) IFN-γ, (e) IL-6 and (f) TNF-α in different treatment groups. (g) Distant tumor and (h) primary tumor weight of mice. (i) Body weight in bilateral 4T1 tumor model mice. Fig. S5. (a) CD4 and CD8 immunofluorescence staining of distant and primary tumor sections. Scale bar: 50 μm. (b) H&E staining of the major organs. Scale bar: 100 μm. (c) Serum biochemical analysis of mice in each treatment group. [file 40824_2023_447_MOESM1_ESM.zip › S3_ESM.pdf]

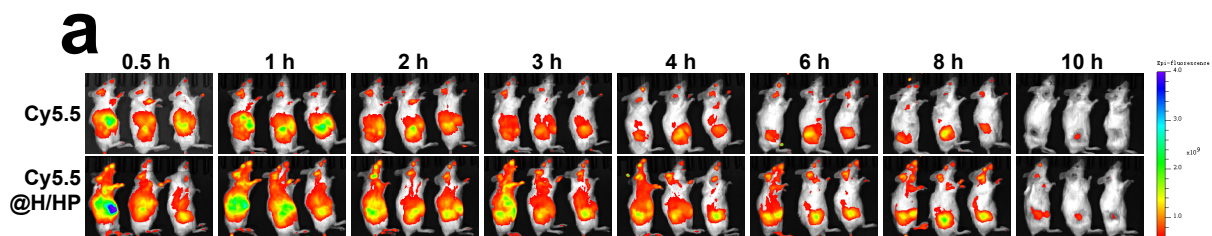

**b**

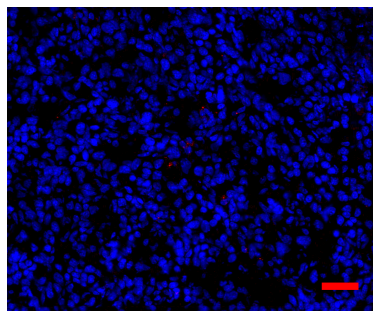

**c**

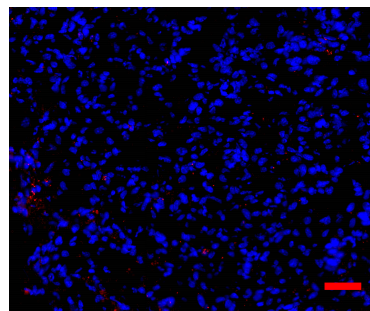

**d**

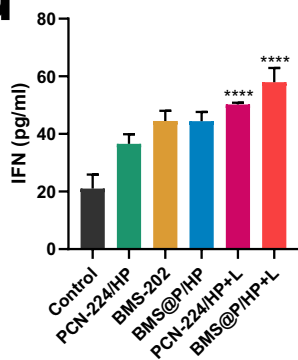

**e**

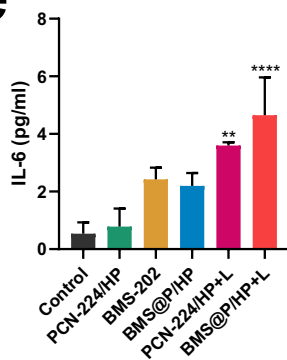

**f**

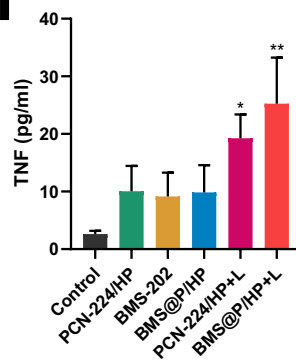

**g**

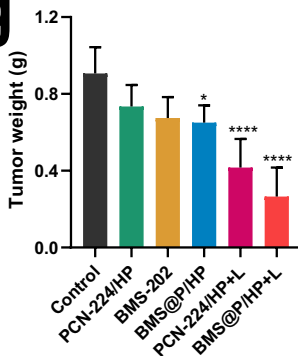

**h**

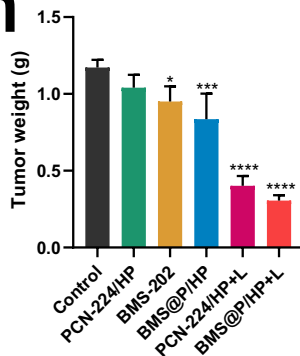

**i**

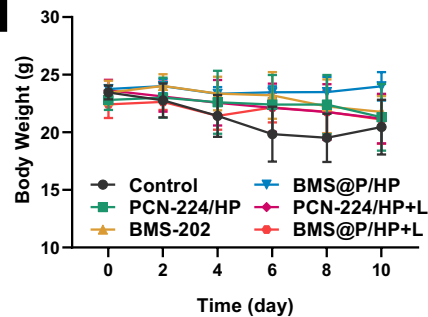

Supplement: Supplementary file 1 — Additional file 1: Fig. S1. (a) The picture of (i) PCN-224, (ii)PNC-224/HP and (iii) BMS@P/HP NPs. (b) TEM image of BMS@PCN-224 NPs. Scale bar: 100nm. (c) Powder X-ray diffraction spectra of PCN-224 NPs. (d) The UV spectrum of TCPP and PCN-224 NPs. (e) FTIR spectrums of PCN-224 NPs. Fig. S2. (a)Synthesis route of HA-PEG. (b) The 1H-NMR spectrum of HA, mPEG-NH2 and HA-PEG. (c) The FTIR spectrum of HA, mPEG-NH2 and HA-PEG. In vitro release profiles of BMS, BMS@PCN-224 and BMS@P/HP at pH (d) 5.5, (e) 6.5 and (f) 7.4. Fig. S3. The uptake of C-6@PCN-224/HP NPs in 4T1 cells was determined by (a) fluorescence microscopy and (b) FCM. (c) Study on colocalization of C-6@PCN-224/HP NPs and lysosomes. Scale bar: 20 μm. (d) Experiments on cell clone clusters treated with different drugs. (e) CRT immunofluorescence staining of 4T1 cells in different treatment groups. Scale bar: 20 μm. Fig. S4. (a) Biodistribution of Cy5.5 and Cy5.5@P/HP NPs in 4T1 tumor bearing mice. Frozen section of tumors in (b) Cy5.5 and (c) Cy5.5@P/HP NPs groups. Tumor cells were stained with blue and the cy5.5 is red. Scale bar: 50 μm. Serum levels of cytokines (d) IFN-γ, (e) IL-6 and (f) TNF-α in different treatment groups. (g) Distant tumor and (h) primary tumor weight of mice. (i) Body weight in bilateral 4T1 tumor model mice. Fig. S5. (a) CD4 and CD8 immunofluorescence staining of distant and primary tumor sections. Scale bar: 50 μm. (b) H&E staining of the major organs. Scale bar: 100 μm. (c) Serum biochemical analysis of mice in each treatment group. [file 40824_2023_447_MOESM1_ESM.zip › S4_ESM.pdf]

**a**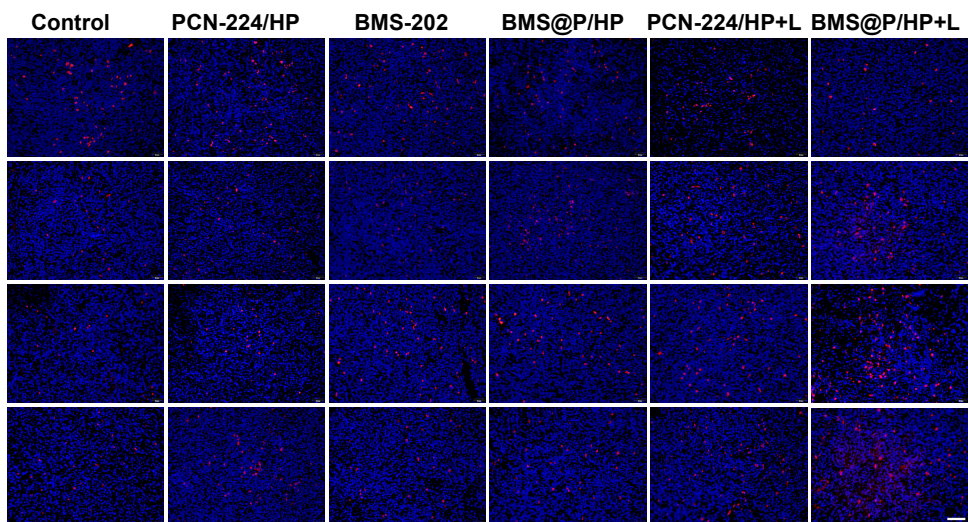**b**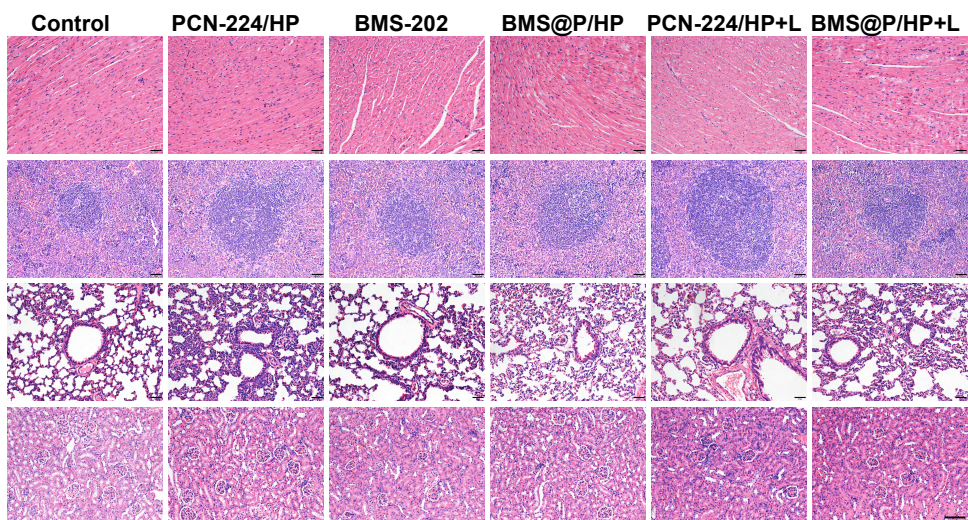**c**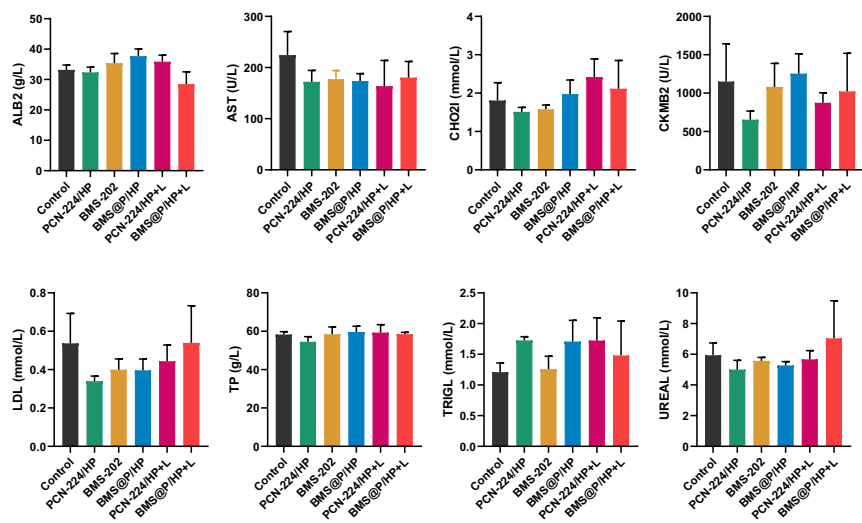

Supplement: Supplementary file 1 — Additional file 1: Fig. S1. (a) The picture of (i) PCN-224, (ii)PNC-224/HP and (iii) BMS@P/HP NPs. (b) TEM image of BMS@PCN-224 NPs. Scale bar: 100nm. (c) Powder X-ray diffraction spectra of PCN-224 NPs. (d) The UV spectrum of TCPP and PCN-224 NPs. (e) FTIR spectrums of PCN-224 NPs. Fig. S2. (a)Synthesis route of HA-PEG. (b) The 1H-NMR spectrum of HA, mPEG-NH2 and HA-PEG. (c) The FTIR spectrum of HA, mPEG-NH2 and HA-PEG. In vitro release profiles of BMS, BMS@PCN-224 and BMS@P/HP at pH (d) 5.5, (e) 6.5 and (f) 7.4. Fig. S3. The uptake of C-6@PCN-224/HP NPs in 4T1 cells was determined by (a) fluorescence microscopy and (b) FCM. (c) Study on colocalization of C-6@PCN-224/HP NPs and lysosomes. Scale bar: 20 μm. (d) Experiments on cell clone clusters treated with different drugs. (e) CRT immunofluorescence staining of 4T1 cells in different treatment groups. Scale bar: 20 μm. Fig. S4. (a) Biodistribution of Cy5.5 and Cy5.5@P/HP NPs in 4T1 tumor bearing mice. Frozen section of tumors in (b) Cy5.5 and (c) Cy5.5@P/HP NPs groups. Tumor cells were stained with blue and the cy5.5 is red. Scale bar: 50 μm. Serum levels of cytokines (d) IFN-γ, (e) IL-6 and (f) TNF-α in different treatment groups. (g) Distant tumor and (h) primary tumor weight of mice. (i) Body weight in bilateral 4T1 tumor model mice. Fig. S5. (a) CD4 and CD8 immunofluorescence staining of distant and primary tumor sections. Scale bar: 50 μm. (b) H&E staining of the major organs. Scale bar: 100 μm. (c) Serum biochemical analysis of mice in each treatment group. [file 40824_2023_447_MOESM1_ESM.zip › S5_ESM.pdf]
